# Residual risk of transfusion-transmitted infection with human immunodeficiency virus, hepatitis C virus, and hepatitis B virus in Korea from 2000 through 2010

**DOI:** 10.1186/1471-2334-12-160

**Published:** 2012-07-20

**Authors:** Moon Jung Kim, Quehn Park, Hyuk Ki Min, Hyun Ok Kim

**Affiliations:** 1 Department of Laboratory Medicine, Yonsei University, College of Medicine, 50 Yonsei-ro, Seodaemun-gu, Seoul, 120-752, South Korea; 2 Chung-Ang University Hospital, Seoul, South Korea; 3 Blood Services Headquarters, Korean Red Cross, Seoul, South Korea

**Keywords:** Residual risk, Transfusion-transmitted infection, Blood safety, Donor screening test

## Abstract

**Background:**

Despite screening blood donations with advanced technologies and improved donor screening, the risk of transfusion-transmitted infections persists. This risk is mainly due to blood donations collected during the window period. A precise estimate of the transfusion risk of viral infection will help to determine the effect of new and current safety measures and to prioritize and allocate limited resources. Therefore, we estimated the risk of transfusion-transmitted viral infection in blood donations collected in Korea from 2000 to 2010.

**Methods:**

Blood donations collected at 16 blood centers were tested for HIV, HCV, and HBV to estimate the residual risk of transfusion-transmitted viral infection. The residual risk was calculated in two-year periods using the incidence/window model. The incidence rates for HIV/HCV and the confirmed positive rate for HIV/HCV in first-time and repeat donors were compared.

**Results:**

The residual risks for HIV in 2004/2005 and 2009/2010 were 1 in 1,080,244 and 1 in 1,356,547, respectively. The risks for HCV in 2000/2001 and 2009/2010 were 1 in 81,431 and 1 in 2,984,415, and the risks for HBV in 2000/2001 and 2009/2010 were 1 in 45,891 and 1 in 43,666. These estimates indicate that the residual risks for HCV in Korea have declined 36.6-fold, and those for HIV and HBV have not improved significantly, compared to previous estimates. The odds ratios for HCV and HBV positivity in first-time donors compared to repeat donors were 11.8 and 19.6, respectively.

**Conclusions:**

The residual risk of HCV declined over the last decade due to improved screening reagents, implementation of the nucleic acid amplification test, and tight application of strict donor selection procedures. Current residual risk estimates for HIV and HCV in Korea are extremely low, but the risk for HBV is still high; therefore, urgent measures should focus on decreasing the residual risk of HBV. Despite the introduction of more sensitive assays in blood screening, several other factors may influence the actual residual risk of transfusion-transmitted infection. A continuous monitoring of residual risk of transfusion-transmitted infection is crucial in managing blood safety.

## Background

The realization that human immunodeficiency virus (HIV) can be transmitted through transfusion mobilized huge public concern for blood safety in the 1980s. Testing donations with advanced technologies and excluding donors at high risk of infection have reduced the risk of infectious donations entering the blood supply. The risk of transfusion-transmitted infection persists, however, mainly due to blood donations collected during the window period.

A precise estimate of the transfusion risk of viral infection will help to monitor transfusion safety, to assess the value of new screening interventions, to analyze the effects of current safety measures, and to assist in developing public health policy. Furthermore, an accurate estimate of residual risk can help inform evidence-based decisions in terms of prioritizing limited resources for hepatitis B virus (HBV), which is more prevalent in Korea than in countries such as the United States, Canada, the UK, and some other European nations.

Current estimates of the residual risks of transfusion-transmitted infection with HIV, hepatitis C virus (HCV), and HBV in Korea are drawn from old studies that only examined donors for short periods of time [1]. Therefore, we estimated the risk of HIV, HCV, and HBV in blood donations collected in Korea between 2000 and 2010. This information may be useful in adapting the national policy for blood transfusion, as well as in deciding among interventions targeted at blood safety.

## Methods

Estimates of the residual risk of transfusion-transmitted viral infection were based on the incidence/window model [2]. All donations collected between January 2000 and December 2010 at the Korean Red Cross Blood Center were included in this study. Data included the number of blood donations, the number of donors who donated blood at least twice during each two-year period, the sum of intervals (in days) between the first and last donation for each donor, the number of donors that made a negative blood donation followed by a positive donation (seroconvertors), the sum of intervals (in days) between the positive and negative donations for seroconvertors, the number of confirmed positive donations, the screening assays, the methods used to confirm donations, and donation history.

Donors were classified as first-time (donors not known to have previously donated blood) or repeat donors (donors who had donated blood before).

Incidence rates were calculated for donors who donated at least twice during each two-year period. The number of incident cases (numerator) was the number of donors who gave a negative donation followed by a confirmed positive blood donation. The window periods for anti-HIV, HIV-NAT, anti-HCV, HCV-NAT, and HBsAg (EIA, CLIA) were obtained from previous reports (Table 1) [3-7]. The incidence rate for each virus was multiplied by the length of the window to calculate the residual risk of infection [4,6-8].

**Table 1:** Length of the window period for test reagents

	**Length of window period (days)**	
	**Estimate**	**Range**
HIV		
anti-HIV(EIA)	22*	6~38
HIV-NAT	11*	
HCV		
anti-HCV(EIA)	66†	38~94
HCV-NAT	10‡	
HBV		
HBsAg(EIA)	59§	37~87
HBsAg(CLIA)	45¶	

*Data were obtained from Busch et al. [3].

†Data were obtained from Courouce et al. [6].

‡Data were obtained from Dodd et al. [7].

§Data were obtained from Mimms et al. [4].

¶Data were obtained from Comanor et al. [5].

All donations were screened for anti-HIV, anti-HCV, and HBsAg using the assays listed in Table 2. Enzyme immunoassays were used for anti-HIV and anti-HCV. Samples were considered confirmed positive for anti-HIV if they were reactive by Western blot and for anti-HCV if they were reactive by immunoblot.

**Table 2:** Donor screening tests for HIV, HCV, and HBV from 2000 to 2010

	**2000**	**2001**	**2002**	**2003**	**2004**	**2005**	**2006**	**2007**	**2008**	**2009**	**2010**
HIV											
anti-HIV (EIA)	DA^*^	DA	DA	GC^†^	GC	LG/GC^‡^	LG/GC	LG/BioRad	LG/BioRad	LG	LG
HIV-NAT						Roche/Chiron	Roche/Chiron	Roche/Chiron	Roche/Chiron	Roche/Chiron	Roche/Chiron
HCV											
anti-HCV (EIA)	LG/SI^§^	LG/SI	LG/SI	LG	LG/DA	SI/LG	LG	LG	LG	LG	LG
HCV-NAT						Roche/Chiron	Roche/Chiron	Roche/Chiron	Roche/Chiron	Roche/Chiron	Roche/Chiron
HBV											
HBsAg (EIA/CLIA)	DA/GC	DA/GC	DA/GC	GC/DA	GC	GC/LG	BioRad/SI	Abbott	Abbott	Abbott	Abbott

^*^DA, Dong-A Pharm.

^†^GC, Green Cross co.

^‡^LG, LG Corp.

^§^SI, Samil co. LTD.

In February 2005, the Korean Red Cross introduced NAT to screen blood donations for HIV-RNA and HCV-RNA. HIV-NAT and HCV-NAT were performed on a minipool of twenty-four samples using the AmpliScreen HIV-1/HCV test (Roche Diagnostics, Branchgurg, NJ, USA) on the Cobas Amplicor, combined with the Organon Nuclisens extractor or on a minipool of sixteen samples using the Procleix HIV-1/HCV assay (Chiron/Gen-Probe, Emeryville, CA, USA). Approximately 18.2 million allogeneic donations were screened for HIV-1 and HCV by the Roche COBAS AmpliScreen assay, whereas the Gen-Probe transcription-mediated amplification system was used to screen approximately 7.8 million donations. RNA was extracted from the plasma pools and screened for HIV-1 RNA and HCV RNA by the COBAS AmpliScreen HIV-1 and HCV test, v2.0. If a 24-member pool tested reactive, further testing was performed on for 6-member secondary pools. If a secondary pool tested reacted, its 6 members were tested individually using the COBAS AmpliScreen HCV test, v2.0. The NAT for HIV-1 and HCV was performed on 16 mini-pool of donor plasma. A repeatedly reactive sample was tested with the HIV-1 and HCV discriminatory assay (Procleix TMA HIV-1/HCV Assay) to identify the type of viral RNA (HIV-1 and/or HCV) present.

Since July 2007, the Korean Red Cross has screened all blood donations using the chemiluminescence immunoassay (CLIA) (Abbott Laboratories, Diagnostic Division, Abbott Park, IL, USA) for HBsAg. Since the implementation of CLIA in July 2007, the number of confirmed HBV cases was estimated from the confirmatory rate (87.5%) of HBsAg among Korean blood donors using a HBsAg neutralization test (PRISM, Abbott Diagnostics, Chicago, IL, USA) [9]. Since the HBV-NAT was not introduced yet, only HBsAg samples with a signal-to-cutoff ratio (S/CO) equal to or greater than 6.00 were considered as confirmed positive to exclude the false positivity of neutralization test [10].

The NAT window period for HIV was used after the introduction of NAT. The window period of anti-HCV was used to calculate the residual risk for HCV until the introduction of HCV-NAT. After the implementation of NAT system, the NAT window period for HCV was used to calculate the residual risk for HCV. The window period of HBsAg enzyme immunoassay was used to calculate the residual risk for HBV until the implementation of CLIA. After the implementation of CLIA, the window period of HBsAg CLIA was used.

In the transition periods, which are 2004/2005 for HIV and HCV and 2006/2007 for HBV, the weighted averages of the two window periods adjusted by length of time were used to calculate the residual risk for HIV, HCV, and HBV.

The number of true seroconvertor was calculated using the positive predictive value of HBsAg (EIA) before the implementation of CLIA. The positive predictive value (PPV) of HBsAg (EIA) was calculated as 25.0% based on the prevalence of HBV among Korean blood donors and the sensitivity of enzyme immunoassay. The residual risk during 2000 and June 2007 was calculated with the number of seroconvertors drawn from PPV. The number of seroconvertors were adjusted by the duration using EIA and CLIA during the transition period.

Data were analyzed using a statistics package SPSS 20.0 (SPSS Inc., Illinois, USA). Poisson regression for trend was used to evaluate the trend of residual risk for HIV, HCV, and HBV across the period. A p-value of less than 0.05 was considered statistically significant.

The total risk was calculated by summing the risks of first-time donors and repeat donors. Each risk was derived by multiplying the incidence rate of cases in each window period. Each risk was then adjusted by the corresponding percentage in all blood donations.

This study was approved by the Institutional Review Board of the Korean Red Cross.

## Results

From January 2000 to December 2010, Korean Red Cross Blood Centers collected a total of 25,931,924 donations. Repeat donors accounted for 20,914,785 donations (80.7%). During the study period, a total of 43 HIV-positive (2004–2009), 139 HCV-positive (2000–2009), 629 HBV-positive donors (2000–2009) were classified as seroconvertors (Table 3). The residual risk for HCV decreased throughout the study period (p = 0.001). The residual risk of HIV was estimated at 1 in 1,080,244 in 2004/2005 and at 1 in 1,813,998 in 2008/2009 (p = 0.745). The residual risk of HCV decreased continuously from 1 in 81,431 in 2002/2001 to 1 in 4,560,879 in 2008/2009, which is extremely low like that of HIV. The residual risk of HBV was estimated at 1 in 67,826 in 2008/2009 (p = 0.885), which is more than 20 times higher than other viruses (Table 3). The incidence rates among first-time and repeat donors were 1.0 and 2.0 for both HIV and HCV (Tables 4 and 5). The most recent (2009/2010) residual risk of transfusion-transmitted infection in Korea was estimated to be 1 in 1,356,547 donations for HIV, 1 in 2,984,415 for HCV, and 1 in 43,666 for HBV (Table 6). The odds ratios for HIV, HCV, and HBV positivity in first-time donors compared to repeat donors in 2010 were 0.8, 11.8, and 19.6, respectively (Table 7).

**Table 3:** Residual risk of transmitting viral infection by transfusing seronegative units donated from 2000 to 2010

	**2000-2001**	**2002-2003**	**2004-2005**	**2006-2007**	**2008-2009**	**p-value of Poisson regression analysis**
HIV						
No. of seroconvertors	-	-	17	10	16	
Incidence rate per 100,000 person-yr	-	-	1.99	1.31	1.83	
(95% CI)*			(0~8.30)	(0~6.51)	(0~7.81)	
Residual risk (per 100,000 donations)	-	-	0.09	0.04	0.06	p = 0.745
(95% CI)			(0~1.43)	(0~0.95)	(0~1.14)	
RR (1 in)	-	-	1,080,244	2,534,406	1,813,998	
HCV						
No. of seroconvertors	64	31	23	14	7	
Incidence rate per 100,000 person-yr	6.80	3.19	2.69	1.83	0.80	
(95% CI)	(0~18.34)	(0~11.00)	(0~10.02)	(0~7.97)	(0~4.76)	
Residual risk (per 100,000 donations)	1.23	0.58	0.28	0.05	0.02	p = 0.001
(95% CI)	(0~6.14)	(0~3.91)	(0~2.65)	(0~1.07)	(0~0.65)	
RR (1 in)	81,431	173,611	357,197	1,991,405	4,560,879	
HBV						
No. of seroconvertors	126	121	105	175	105	
Incidence rate per 100,000 person-yr	13.48	12.49	12.30	22.87	11.96	
(95% CI)	(0~29.73)	(0~27.95)	(0~27.98)	(1.16~44.58)	(0~27.25)	
Residual risk (per 100,000 donations)	2.18	2.20	1.99	3.48	1.47	p = 0.885
(95% CI)	(0~8.72)	0~8.69)	(0~8.30)	(0~11.95)	(0~6.83)	
RR (1 in)	45,891	49,515	50,218	28,754	67,826	

*CI denotes confidence interval.

**Table 4:** Incidence rate of HIV among first-time donors and repeat donors

	**Incidence rate per 100,000 donations**		
**Period**	**First-time donors**	**Repeat donors**	**Total**
2005-2006	1.0	2.1	1.2
2006-2007	0.6	1.3	0.8
2007-2008	0.7	1.5	0.9
2008-2009	0.9	1.8	1.1
2009-2010	1.2	2.4	1.4
Total	1.0	2.0	1.8

**Table 5:** Incidence rate of HCV among first-time donors and repeat donors

	**Incidence rate per 100,000 donations**		
**Period**	**First-time donors**	**Repeat donors**	**Total**
2005-2006	1.7	3.3	2.0
2006-2007	0.9	1.8	1.1
2007-2008	0.7	1.4	0.8
2008-2009	0.4	0.8	0.5
2009-2010	0.6	1.2	0.7
Total	1.0	2.0	1.8

**Table 6:** Most recent residual risk of transfusion-transmitted infection, 2009 to 2010

	**HIV**	**HCV**	**HBV**
No. of seroconvertors	22	11	167
Incidence rate per 100,000 person-yrs.	2.45	1.96	18.58
Residual risk per 100,000 donations	0.07	0.03	2.29
(95% CI)	(0~1.13)	(0~0.79)	(0~8.90)
Residual risk (1 in )	1,356,547	2,984,415	43,666

**Table 7:** Confirmed positive rate per 100,000 first-time and repeat donors with HIV and HCV and repeat reactive rate per 100,000 donors with HBV

		**2006**	**2007**	**2008**	**2009**	**2010**
HIV	First-time donor	1	3	2	1	1
	Repeat donor	2	2	2	1	2
	Odds ratio	0.4	1.8	1.4	0.8	0.8
HCV	First-time donor	37	32	28	27	27
	Repeat donor	5	4	3	3	2
	Odds ratio	7.1	8.3	9.2	10.9	11.8
HBV	First-time donor	672	588	512	451	396
	Repeat donor	48	30	13	11	20
	Odds ratio	14.0	19.3	38.6	41.4	19.6
Total	First-time donor	710	622	542	479	424
	Repeat donor	56	36	18	15	24
	Odds ratio	12.8	17.4	29.9	32.2	17.5

## Discussion

A residual risk of transfusion-transmitted infection persists despite the adoption of stricter donor selection criteria and continuous improvements in the performance of screening assays. This risk is mainly linked to donations in the window following a recent, undetected infection. The residual risk of transmitting HIV, HCV, or HBV during a blood transfusion was estimated between 2000 and 2010. An incidence/window period model was used to estimate the residual risk. A mathematical model to quantify viral nucleic acid concentrations was developed in the early 2000s, when most developed countries began nucleic acid testing (NAT) for donations [11]. NAT for HIV and HCV in Korea was commenced in February 2005. 10 HIV and 19 HCV NAT yield donations were identified between 2005 and 2010 among 14,246,270 donations. Since the average yield per year was extremely low, the method of Schreiber was used in this study even though it may over-estimate the incidence density.

Residual risk estimates vary among countries, depending on the incidence rates among blood donors and the tests used. Thus, some variations might reflect differences in the incidence and background prevalence, as well as the testing methods used. NAT, which was introduced to industrialized countries in the 1990s and 2000s, dropped the residual risk from 1 in 513,000 to 1 in 3,415,000 for HIV and from 1 in 149,000 to 1 in 1,935,000 for HCV [7,12-14]. The residual risks of HIV and HCV for several countries have become immeasurably small in recent years (approximately 1 infection per 1 to 2 million units) [15,16].

The HBV prevalence differs by geography [17]. The residual risk of HBV in countries with low prevalence (<2%), such as the UK, was 1 in 296,736 (3.37 per million donations) in the 1990s, and 1 in 729,927 (1.37 per million donations) in the 2000s [18]. The residual risk of HBV in countries with moderate to high prevalence declined from 1 in 10,700 to 1 in 340,000 during a similar period [19,20]. Even though the probability of missing a donation infected with HBV has declined over time, the residual risk of HBV is greater than the risks for HIV and HCV.

In this report, we determined the trend of incidence rates and residual risks of infection in donations made to Korean Blood Services through 2000 to 2010. The risk of HCV in 2000/2001 decreased 56-fold by 2008/2009, but those of HIV and HBV have not changed.

The residual risk for HIV did not change significantly. It is due to the fact that the assay for HIV did not change from 2005 to 2010, and the exclusion of donors with high risk behaviors were not done completely. The current risk level of HIV is comparable to developed countries. If the risk of HIV needs to be reduced, other safety measures should be applied to completely exclude the donors with risk behaviors. Blood safety regarding HCV has improved immensely during the last decade due to the implementation of NAT and improved performance of serologic assays. The residual risk has decreased 31% (95% CI, 20~56%) annually (data not shown).

Even though the residual risk for HBV in 2008/2009 has decreased 19.9-fold from that in 1995/1996 [21], the risk for HBV did not change significantly during the last decade. Even after using a more sensitive CLIA, the residual risk of HBV is still substantial. This can be generally explained by differences in infectivity, viral doubling time, and minimum infectious dose. Practically, the reason of the immaterial reduction of risk through the application of CLIA is due to a comparatively small reduction of window period. The reduction rate of window period was only 23.7% and the reduction of residual risk was 32.3% from 2000/2001 to 2008/2009. The current residual risk for HBV is more than 20-fold higher than that for HIV and HCV, and approximately 5-fold higher than those in other countries with moderate to high prevalence. That is why the introduction of HBV-NAT is urgent in Korea and will commence in July 2012 nationwide. The residual risk of HBV will be efficiently reduced through an individual HBV-NAT like Spain, which has a similar prevalence of HBV [22]. However, HBsAg will remain as one of the mandatory tests for blood screening after the implementation of HBV-NAT, since Korea is an endemic area and dropping a blood screening test is not an easy decision for governmental officials.

Interestingly, the serial trend of residual risk showed that the residual risks of all three viruses increased in 2009/2010 compared to the previous study period (2008/2009). This phenomenon might be attributed to the application of several strong incentive systems for blood donors commenced in 2010 to secure the blood supply. Approximately 80% of Korean blood donors are in their 10s and 20s. The target population of donor recruitment programs is usually young students. The Korean parents are excessively education-oriented, and the students are extremely sensitive to the incentives related to extra credit for school. In this situation, application of donor recruitment related to the school grade might hinder the effective exclusion of donors with high risk behaviors. Therefore, incentives especially for young donors should be applied with discretion. Generally the residual risk depends upon the prevalence and the sensitivity of the test method (window period of the test). However, other factors besides these two traditional typical factors could influence the actual residual risk. Therefore, a continuous monitoring of residual risk is critical in management of blood safety, even though the same test method is continually used and the prevalence of disease does not change. Even though the declining trend of residual risk for HCV has stopped and increased in 2009/2010 and residual risk for HIV in 2009/2010 was higher than that in 2008/2009, the current residual risks for HIV and HCV do not substantially differ from those of developed countries.

The assays have changed several times over the years that data were analyzed. NAT was commenced in 2005 and the same reagents and the same instruments have been used until 2010. Since the assay reagents and instruments were maintained the same for more than 70-80% of blood donations, the change of assays does not seem to affect the estimates significantly.

We adjusted the total residual risk for the incidence of first-time donors. This estimate was based on the fact that donor behavior was not affected during the window period, but donors with an early acute infection with subtle mild symptoms or a history of risky behavior might delay the next donation. Thus, the residual risk might be underestimated due to an inaccurate and smaller number of seroconvertors among blood donors.

The incidence rates of HCV and HBV were higher among first-time donors than repeat donors. The incidence rates of HIV did not differ among first-time and repeat donors. Some repeat donors with high-risk behavior may not have answered the questionnaire honestly. Our HIV findings differed from previous reports [16,23].

We did not include donors with chronic infection in the risk assessment. Even though the infectivity from donors with a chronic hepatitis C infection is 1,000 times weaker than from donors with an acute hepatitis C infection, the donation from a donor with chronic HCV can be infectious [24]. Likewise, low-level HBV carriers and HBsAg mutant viruses were not considered in our estimate of residual risk.

Our model does not reflect infectivity. A minimum infectious dose might cause infectivity to differ during the window period, but all donations during the window period were assumed to be infectious.

Current data suggest that HIV is considerably less infectious than HCV and HBV. A recent report shows that 100 to 10,000 copies of transmitted HIV RNA falls well in the 0- to 100-percent infectivity range [25]. Literature shows that less than 100 copies of HCV and HBV can cause infection [26].

Recently, Busch et al introduced a new approach to estimate the duration of infectious window period, based on back-extrapolation of acute viral replication dynamics [27]. The NAT yield data for the last 6-year period seemed to be large enough to apply an alternative method, but the estimated window period of HCV NAT was too long to accept. As Busch et al mentioned about their limitation, the new method was based on the concept that the incidence in donor population does not change significantly, which was not fit in our situation. There are multiple factors that could influence the residual risk other than the prevalence and the sensitivity of screening assay. This phenomenon seems to be attributed to the unique cultural environment in Korea, such as application of excessive pre-cautionary safety measures after the blood scandal in 2005. Using NAT screening data from large numbers of donations by a new approach did not always guarantee an accurate estimation of window period.

We may have overestimated the residual risk by assuming that all window period donations were infectious. The presence of virus in a donation does not imply that the recipient was infected. The viral load may have been too low, the infectivity may have been too weak, or the recipient might have been vaccinated.

Korea has a moderate to high risk of HBV [28]. HBsAg-positive rate reached 4.6% in Korean residents aged 10 and older in 1998, and the prevalence dropped gradually to 2.9% in 2010 [29]. However, the prevalence of HBV is still substantially higher than those in other developed countries. Since introducing the hepatitis B virus vaccine in 1985, the prevalence of immunized residents aged 30 and older has reached 58.0%. One study showed that the positive rate of anti-HBs was approximately 80% in young patients [30]. The universal vaccination of HBV since 1995 will contribute to the reduction of residual risk of HBV in Korea. Thus, the actual transmission of HBV from a transfusion is likely lower than the estimated residual risk.

## Conclusion

The residual risk of HCV declined over the last decade due to improved screening reagents, implementation of the nucleic acid amplification test, and tight application of strict donor selection procedures. Current residual risk estimates for HIV and HCV in Korea are extremely low, but the risk for HBV is still high; therefore, urgent measures should focus on decreasing the residual risk of HBV. Despite the introduction of more sensitive assays in blood screening, several other factors may influence the actual residual risk of transfusion-transmitted infection. A continuous monitoring of residual risk of transfusion transmitted infection is required to manage blood safety.

## Competing interests

All authors declare that they have no competing interests.

## Authors’ contributions

MK and HM were involved in the study design. MK and QP were involved in analysis and interpretation of the data. HK reviewed the initial and final drafts of the manuscript. All authors read and approved the final manuscript.

## Pre-publication history

The pre-publication history for this paper can be accessed here:

http://www.biomedcentral.com/1471-2334/12/160/prepub
